# A diverse assemblage of *Ptychodus* species (Elasmobranchii: Ptychodontidae) from the Upper Cretaceous of Ukraine, with comments on possible diversification drivers during the Cenomanian

**DOI:** 10.1016/j.cretres.2023.105659

**Published:** 2023-11

**Authors:** Manuel Amadori, Oleksandr Kovalchuk, Zoltán Barkaszi, Luca Giusberti, René Kindlimann, Jürgen Kriwet

**Affiliations:** a Department of Palaeontology, University of Vienna, Josef-Holaubek-Platz 2, 1090, Vienna, Austria; b Department of Palaeontology, National Museum of Natural History, National Academy of Sciences of Ukraine, 15 Bohdan Khmelnytskyi Street, Kyiv 01054, Ukraine; c Department of Palaeozoology, Faculty of Biological Sciences, University of Wrocław, 21 Sienkiewicza Street, Wrocław 50-335, Poland; d Dipartimento di Geoscienze, Università degli Studi di Padova, Via Gradenigo 6, I-35131, Padova, Italy; e Sammlung R. Kindlimann, Aathal-Seegrӓben, Switzerland; f Vienna Doctoral School of Ecology and Evolution (VDSEE), University of Vienna, Djerassiplatz 1, 1030 Vienna, Austria

**Keywords:** Chondrichthyes, Taxonomy, Paleoecology, Epicontinental Sea, Niche partitioning, Eastern Europe

## Abstract

New isolated teeth from the Upper Cretaceous of Ukraine and belonging to the extinct durophagous shark *Ptychodus* are described here. The taxonomic identification of the examined material reveals a quite diverse Cenomanian shark fauna which comprised both cuspidate and un-cuspidate species of *Ptychodus* from the coastal areas at the north-western margin of the Ukrainian Massif. In addition, *P. latissimus* from the Turonian of Ukraine is reported here for the first time. The revision of the Ukrainian record of *Ptychodus* revealed that most specimens described here are the oldest so far known from this part of the European Peri-Tethys. Moreover, the present study highlights the co-occurrence of cuspidate and un-cuspidate *Ptychodus* and a variety of shelled macroinvertebrates, which inhabited coastal and offshore areas of the European epicontinental seas during the Late Cretaceous. The availability of different prey items is proposed here as one of the possible drivers, in addition to abiotic environmental factors, for the diversification of shark tooth morphologies, and possible trophic partitioning between cuspidate and un-cuspidate species of the genus *Ptychodus*.

## 1 Introduction

Modern sharks, together with their relatives (rays and skates), play crucial roles in trophic marine food webs and thus have important structural and functional influence on oceanic ecosystems (Duffy et al., 2015; Bizzarro et al., 2017; Yokoi et al., 2017; Barkaszi and Kovalchuk, 2021; Dulvy et al., 2021; Kovalchuk and Barkaszi, 2021). However, shark populations, especially those in pelagic environments, have dramatically decreased in recent decades due to the increasing global fisheries and climate change, threatening these marine predators with extinction, because they only slowly recover from overexploitation (Baum et al., 2003; Ferretti et al., 2008; García et al., 2008; Dulvy et al., 2021). Consequently, appropriate conservation and management measures of shark faunas have become vital to protect both marine biodiversity and oceanic ecosystem functioning (Barker and Schluessel, 2005; Heithaus et al., 2008; Yokoi et al., 2017; Marongiu, 2023).

Evaluation of organismal responses to biotic and abiotic factors in deep time can provide valuable insights into past, current and future diversity drivers and sustainers as well as new perspectives of our understanding of future marine community dynamics (e.g., Jablonski and Shubin, 2015). Climatic and environmental changes influence directly the diversity of sharks, rays, and skates, the Elasmobranchii *sensu* Maisey (2012) either through variations in prey and habitat availability or distribution patterns (Heupel and Hueter, 2002; Torres et al., 2006). Therefore, better understanding of changes in the structure and functioning of marine ecosystems and their impact on elasmobranchs may also be possible by studying their diet adaptations (Plumlee and Wells, 2016). Although dental morphologies are not always predictive of predator biological roles (Whitenack and Motta, 2010), peculiar dental features and tooth arrangements can be helpful to infer possible feeding specializations and fairly identify the main prey range of various groups of extant and extinct elasmobranchs (e.g., Walker and Brett, 2002; Ciampaglio et al., 2005; Shimada, 2012; Crofts and Summers, 2014; Corn et al., 2016; Amadori et al., 2019b, 2020a,b, 2022a; Hamm, 2020).

Elasmobranchs are commonly considered to be opportunistic predators being not strictly selective in their dietary range (e.g., Huber et al., 2019). However, defining all sharks indiscriminately as generalists may be an oversimplification of their complex trophic ecology (e.g., Summers et al., 2004; Wetherbee et al., 2012; Moyer and Bemis, 2017; Huber et al., 2019; Bazzi et al., 2021). Among many trophic specializations that have evolved in elasmobranchs, the ability to crush shelled prey (durophagy) was acquired several times independently and through multiple adaptations during long and successful evolutionary history of this group (Summers et al., 2004; Huber et al., 2005; Kolmann et al., 2015; Herbert and Motta, 2018). Durophagous sharks often combine massive jaws equipped with molariform teeth, extremely well-developed adductor muscles and very force-efficient jaw leverage resulting in high bite forces to successfully break armored prey (Nobiling, 1977; Summers, 2000; Wilga and Motta, 2000; Huber et al., 2005). Molariform teeth and corresponding occlusal dental wear patterns found in extinct elasmobranch species are usually correlated with the ability of crushing shelled prey (Woodward, 1912; Shimada, 2012; Amadori et al., 2019a,b, 2020a,b, 2022a; Niebuhr and Wilmsen, 2022).

Durophagous sharks seemingly were more diverse in the past as exemplified by their fossil record, which include numerous teeth (e.g., Cappetta, 2012). Among them, one of the most speciose is the enigmatic, extinct shark *Ptychodus*. Its remains have been recovered from Cretaceous deposits of all continents except Antarctica (Woodward, 1912; Herman, 1977; Siverson, 1999; Cappetta, 2012; Hamm, 2020; Amadori et al., 2022b). Within the genus *Ptychodus*, two groups characterized by cuspidate and un-cuspidate teeth respectively can be distinguished based on tooth morphologies (e.g., Cappetta, 2012; Shimada, 2012; Amadori et al., 2019a,b, 2020a,b; Hamm, 2020). These durophagous elasmobranchs mostly occurred in epicontinental seas during the Cretaceous and they might have been capable of both feeding on the seabed and hunting within the water column (Everhart, 2017; Hamm, 2020; Amadori et al., 2020a). Moreover, an opportunistic feeding strategy was also hypothesized at least for some cuspidate taxa, such as *P. altior, P. maghrebianus* and *P. occidentalis* (Shimada et al., 2009; Amadori et al., 2019b, 2022a). Although the un-cuspidate *P. decurrens* represents the oldest species of the genus, the causes and timing for the diversification of un-cuspidate taxa with heavily ornamented teeth (e.g., *P. latissimus*), as well as various cuspidate forms (e.g., *P. altior, P. mammillaris, P. mortoni, P. rugosus*, and *P. whipplei*), remain unclear and doubtful (Cappetta, 2012; Shimada, 2012; Hamm, 2020; Amadori, 2022).

In this paper, we describe a sample of newly discovered isolated teeth of various *Ptychodus* species from Cenomanian and Turonian deposits of Ukraine. We also provide a general summary of the Upper Cretaceous record of *Ptychodus* within this region. In addition, the taxonomic composition and tooth morphologies of European *Ptychodus* species are discussed herein in a paleoenvironmental context focusing on epicontinental environments of the Late Cretaceous seas. Finally, we propose the availability of different prey items (e.g., ammonites, bivalves, and decapods) in epicontinental areas across Europe during the Cenomanian as one of the possible drivers for the diversification of tooth morphologies within the genus *Ptychodus*.

## 2 Geological setting

The studied material comes from a few localities within the north-eastern part of the Ukrainian Shield and northern part of the VolynePodolian Plate. During the Late Cretaceous, the sea continued to expand in the territory of modern Ukraine, although it was relatively shallow. Sands, sandstones, marlstones, limestones, and chalk evidenced the paleogeographical setting of this epicontinental basin (Ivanik et al., 2013). Low islands were located in some areas of the Ukrainian Shield, Donets Basin, and the Pre-Carpathian fault (Kraeva et al., 1960a; Ivanik et al., 2013; Kyselevych and Kovalchuk, 2021).

The Cenomanian basin that existed in the territory of modern Ukraine had a wide connection with the West European Basin through the Prypiat Fault and modern Poland, as well as with the Mediterranean Basin through the Crimea–Caucasian region (Kraeva et al., 1960a). Cenomanian deposits in Ukraine are exclusively marine and display mainly uniform lithological composition. Sands and sandstones with phosphorite pebbles characterize the lower Cenomanian, whereas sandy chalks, marlstones, and limestones constitute upper Cenomanian deposits (Plotnikova, 1971; Ivanik et al., 2013).

**Fig. 1:**
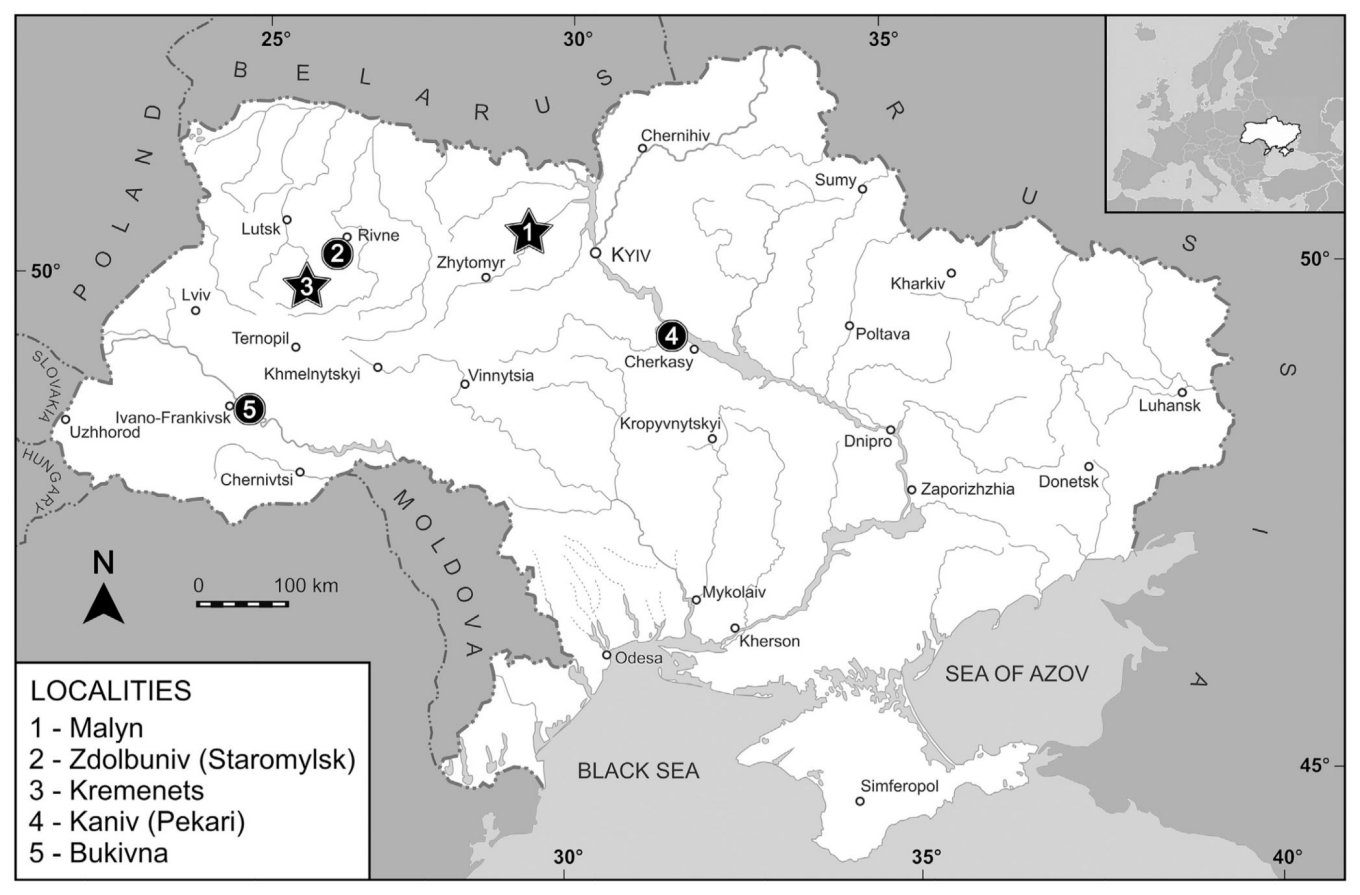
Location map of the Ukrainian sites (black dots) from where *Ptychodus* has been hitherto reported. The black stars indicate the localities from which the material examined in the present study came.

Most of the specimens described herein were collected in an artificial outcrop near Malyn (see Fig. 1), west of Kyiv, in a quarry on the second fluvial terrace of the Irsha River (Bukovich et al., 1976). Marine deposits in this section are represented by shallow coastal facies (Kyselevych and Ogienko, 2018). The Cenomanian layer is 1.5 m thick, and it is represented by detrital limestone consisting of shell fragments with grains of glauconite (Bukovich et al., 1976). The teeth of *Ptychodus* were recovered together with those of lamniform sharks, teleost fishes (Kovalchuk et al., 2022) and ichthyosaurs (Kyselevych and Ogienko, 2018). Rogovich (1861) previously recovered and briefly described a set of isolated teeth of *Ptychodus* from Cenomanian green sandstones exposed near the village of Pekari in the vicinities of Kaniv (central part of Ukraine; see Fig. 1).

Marine transgressive phases continued during the Turonian and Coniacian in the territory of modern Ukraine, and the basin constantly deepened resulting in a gradual change in the nature of deposits, which are dominated today by white chalks with flint concretions, chalky limestones, and marls (Kraeva et al., 1960b; Ivanik et al., 2013).

Teeth of *Ptychodus* rarely occur in black flint concretions within a white chalk layer exposed near Kremenets, western Ukraine (Shakh and Karpenchuk, 1964; Fig. 1). The late Turonian age of these deposits is based on foraminifera, ostracods, mollusks, and sea urchins (Kotsiubynsky and Gynda, 1966; Rozumeiko, 1969; Pasternak, 1971; Didenko, 2005; Matveev et al., 2017). Shakh and Karpenchuk (1964) also reported the presence of scarce *Ptychodus* remains in the Turonian deposits of Staromylsk (Fig. 1).

Seven complete and 13 fragmentary teeth of *Ptychodus* were found 2 km north-west of the village of Bukivna (Fig. 1) by A. Denysevych and Y. Karpenchuk in a 6–7 m thick outcrop of light grey layered chalky limestones, on the right bank of the Dniester River (western Ukraine; see Shakh and Karpenchuk, 1964). In addition to shark teeth, a fragment of *Inoceramus involutus* and diverse small benthic foraminifera (*Spiroplectammina praelonga, Gaudryina tricarinata, Vulvulina reussi, Arenobulimina subsphaerica, A*. aff. *orbignyi, A. senonica, Ataxophragmium ammonoides, A. initiale, Anomalina infrasantonica, A. costulata*, and *Bulimina reussi*) were found in the same layer indicating a Coniacian age for these rocks (Shakh and Karpenchuk, 1964).

## 3 Materials and methods

### 3.1 Institutional abbreviations

NMNHU, National Museum of Natural History of the National Academy of Sciences of Ukraine (NMNHU-G, Department of Geology; NMNHU-P, Department of Palaeontology, collections PI, EX); PIMUZ, Palaeontological Institute and Museum of the University of Zurich (Switzerland).

### 3.2 Materials

The material documented in this study consists of 19 teeth recovered from Kremenets (NMNHU-G 391/15), Kaniv (NMNHU-P EX 1729/1, 1729/2) as well as from the outskirts of Malyn (NMNHU-P PI 1729/1e5, 2342e2352). These specimens are housed in the collections of the National Museum of Natural History of the National Academy of Sciences of Ukraine (NMNHU) in Kyiv. In addition, four teeth (catalogue numbers PIMUZ A/I 5234-5237) from Malyn are documented here. These additional specimens are in the collection of the Palaeontological Institute and Museum of the University of Zurich (Switzerland) and they are currently on display in the “Haimuseum und Sammlung R. Kindlimann” in Aathal-Seegräben (Switzerland), with public access. Table S1 (online supplementary data) provides more details on the material described here.

### 3.3 Methods

#### 3.3.1 General procedures

The material from Malyn was collected by O. Kovalchuk and Z. Barkaszi in 2021 using dry screening of about 50 kg of rocks through a 5 mm sieve. The specimen embedded in black flint concretion from Kremenets was obtained by O. Rogovich from the white chalk layer in 1844. Two isolated teeth from natural exposures near Kaniv were originally collected in the 1970s. The specimens were photographed using Nokia 7 Plus camera (ZEISS optics) and a 25 mm lens. Illustrative drawings, maps and images of the specimens were prepared using the Photoshop CS6 (v. 23.2.1) software package.

#### 3.3.2 Data

Geographic and stratigraphic occurrences of *Ptychodus* are based on Amadori et al. (2022b) and updated with the Ukrainian records reported in the present paper. Data on the occurrences of the possible prey of *Ptychodus* (ammonites, inoceramids and decapods) were obtained from the Paleobiology Database (PaleoDB, https://paleobiodb.org/ downloaded on 13/03/2023; see also Uhen, 2018) and updated when possible (see also Table S2 in online supplementary data).

#### 3.3.3 Terminology and nomenclature

The morphological terminology used herein mostly follows Cappetta (2012) and Amadori et al. (2019b, 2020a). The open nomenclature adopted herein follows the standards proposed by Bengtson (1988) and Sigovini et al. (2016).

## 4 Results

### 4.1 Systematic paleontology

Class: Chondrichthyes Huxley, 1880

Subclass: Elasmobranchii Bonaparte, 1838

Order: Ptychodontiformes Hamm, 2019

Family: Ptychodontidae Jaekel, 1898

Genus *Ptychodus* Agassiz, 1834

*Type species. Ptychodus schlotheimii* Agassiz, 1834 (*nomen oblitum*), senior synonym of *Ptychodus latissimus* Agassiz, 1835 (*nomen protectum*). See Giusberti et al. (2018).

*Diagnosis*. See Woodward (1912).

***Ptychodus altior*** Agassiz, 1835

(Fig. 2A-A^III^)

For synonyms, see Amadori et al. (2019b).

*Diagnosis*. See Amadori et al. (2019b: p. 5).

*Referred material*. A single tooth, NMNHU-P PI 2351, from Malyn.

**Fig. 2:**
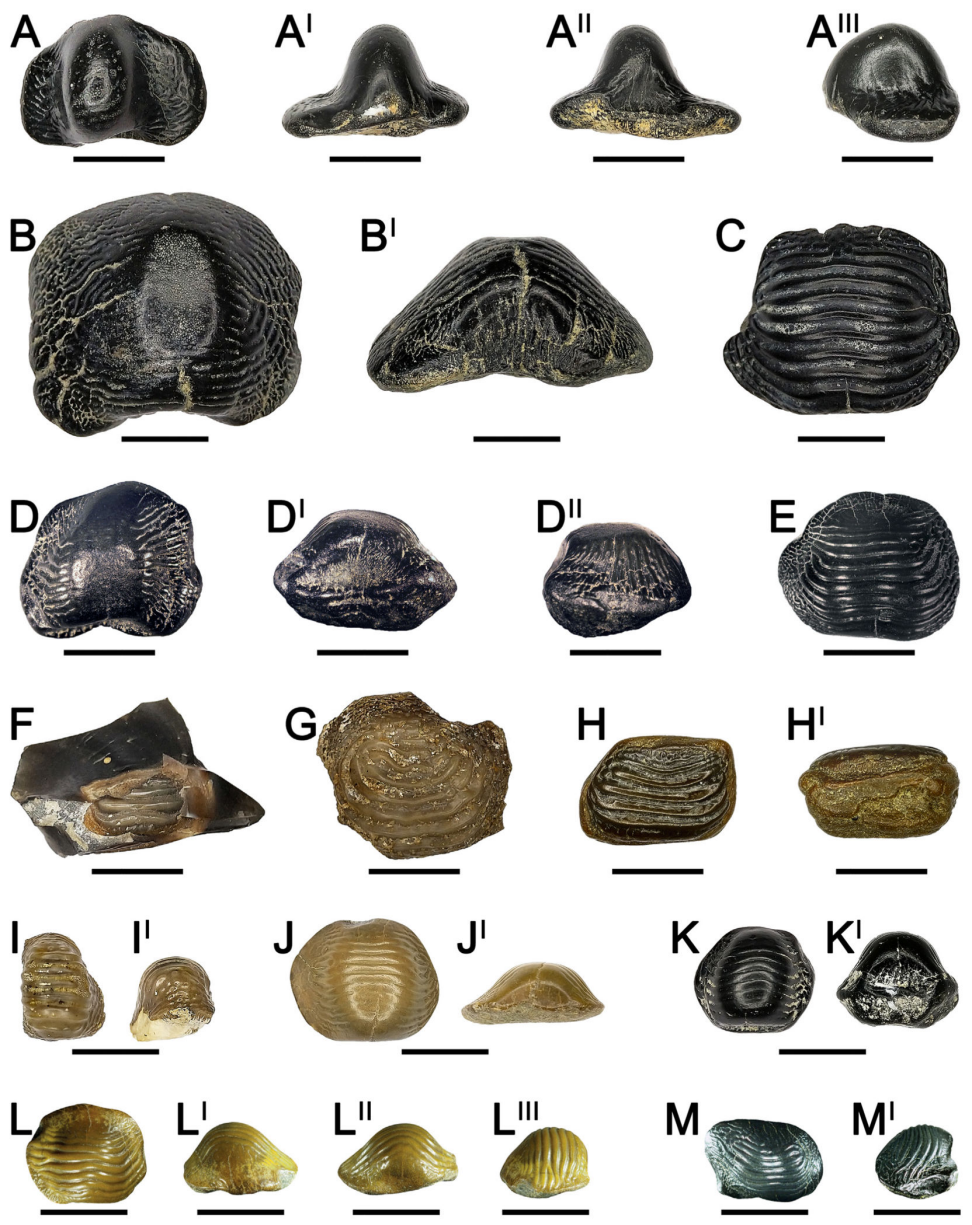
Isolated teeth of *Ptychodus* spp. from Ukraine in occlusal (A, B, C, D, E, F, G, H, I, J and K), anterior (A^I^ and I^I^), posterior (A^II^, B^I^, D^I^, H^I^, J^I^ and K^I^) and lateral (A^III^ and D^II^) views. A-A^III^, tooth NMNHU-P PI 2351 belonging to *P. altior*; B-B^I^, tooth NMNHU-P PI 2342 belonging to *P. decurrens*; C, tooth NMNHU-P PI 2344 assigned herein to †*P*. cf. *marginalis*; D-D^II^, tooth PIMUZ A/I 5235 belonging to *P. decurrens*; E, tooth PIMUZ A/I 5234 belonging to *P. polygyrus*; F, tooth NMNHU-G 391/15 belonging to *P. latissimus*; G, tooth NMNHU-P EX 1729/1 belonging to *P. marginalis*; H-H^I^, tooth NMNHU-P PI 2346 belonging to *P. polygyrus*; I-I^I^, tooth NMNHU-P EX 1729/2 assigned herein to *P*. cf. *anonymus*; J-J^I^, tooth NMNHU-P PI 1729/1 assigned herein to *P*. cf. *mammillaris*; K-K^I^, tooth NMNHU-P PI 2350 assigned herein to *P*. cf. *mammillaris*; L-L^III^, tooth PIMUZ A/I 5236 belonging to *P. decurrens*; M-M^I^, tooth PIMUZ A/I 5237 belonging to *P. decurrens*. Scale bars equal 10 mm.

*Description*. NMNHU-P PI 2351 (Fig. 2A-A^III^) has a thin, asymmetrical dental crown with two lateral lobes, which are curved posteriorly. In anterior view (Fig. 2A^I^), the right lobe is larger than the left one. Both anterior protuberance and posterior sulcus are well-developed (see Fig. A-A^II^). A narrow and rounded cusp characterizes the center of the crown (see Figs. 2A^I^-A^II^). The cusp apex is markedly abraded, and its occlusal ornamentations are barely observable (see Fig. 2A). In occlusal view (Fig. 2A), two lobes (marginal areas) exhibit thin wrinkles curving anteriorly. In lateral view (Fig. 2A^III^), the sides of the dental cusp are smooth. The root is not preserved (see Fig. 2A^I^-A^III^).

*Remarks*. The asymmetrical outline of NMNHU-P PI 2351 (Fig. 2A) supports its assignment to the lateral area of the dentition.

***Ptychodus decurrens*** Agassiz, 1838

(Figs. 2B,B^I^, 2D-D^II^, 2L-L^III^, 2M,M^I^)

For synonyms, see Amadori et al. (2019a).

*Diagnosis*. See Hamm (2020: p. 14).

*Referred material*. Four teeth, NMNHU-P PI 2342 and PIMUZ A/I 5235-5237, from Malyn.

*Description*. NMNHU-P PI 2342 (Fig. 2B,B^I^), PIMUZ A/I 5235 (Fig. 2D-D^II^), PIMUZ A/I 5236 (Fig. 2L-L^III^) and PIMUZ A/I 5237 (Fig. 2M,M^I^) have thick and asymmetric crowns with a deep posterior sulcus. The tooth crown margins are partially damaged in NMNHU-P PI 2342 and PIMUZ A/I 5235, 5236. In occlusal view, their surface is crossed by 7 to fourteen ridges, which are branched distally to the lateral marginal areas of the crown. The ridges in the right marginal area of the NMNHU-P PI 2342 (Fig. 2B) and PIMUZ A/I 5237 (Fig. 2M) curves, forming almost a circular pattern. The ridges are markedly abraded at the center of the crown of NMNHU-P PI 2342 (Fig. 2B) and PIMUZ A/I 5235 (Fig. 2D). In posterior and lateral views (Fig. 2D^I^,D^II^), their crowns are bulged; the roots are missing.

*Remarks*. The asymmetric occlusal outline of NMNHU-P PI 2342 (Fig. 2B), PIMUZ A/I 5235 (Fig. 2D), PIMUZ A/I 5236 (Fig. 2L) and PIMUZ A/I 5237 (Fig. 2M), together with their centrally elevated crowns, suggests that they were originally positioned within the lateral part of the lower dentition. Distally branched, thin ridges are typical for the un-cuspidate *Ptychodus decurrens*. However, the ridges in this species always reach the lateral tooth edges (see Woodward 1912; Amadori et al., 2019a; Hamm, 2020). Circular ridges similar to those on the right marginal areas of NMNHU-P PI 2342 (Fig. 2B) and PIMUZ A/I 5237 (Fig. 2M) are unusual for *P. decurrens*. However, such ornamentation patterns can rarely occur in lateral teeth of this species (e.g., Woodward, 1912: pl.51, fig. 1).

***Ptychodus latissimus*** Agassiz, 1835 (Fig. 2F)

For synonyms, see Amadori et al. (2020a: p. 4).

*Diagnosis*. See Amadori et al. (2020a: p. 6).

*Referred material*. A single tooth, NMNHU-G 391/15, from Kremenets.

*Description*. NMNHU-G 391/15 (Fig. 2F) is an incomplete, rectangular tooth, which still is embedded in a black flint. The anterior side of the crown is missing. In occlusal view (Fig. 2F), four thick and sharp ridges characterize the center of the tooth crown, while coarse granulations cover its marginal areas.

*Remarks*. The fragmentary nature of the specimen prevents establishing its original position within the tooth plate.

***Ptychodus marginalis*** Agassiz, 1839 (*sensu* Hamm, 2020) (Fig. 2G)

For synonyms, see Hamm (2020: p. 24).

*Diagnosis*. See Amadori et al. (2020a: p. 8).

*Referred material*. A single tooth, NMNHU-P EX 1729/1, from Kaniv. *Description*. NMNHU-P EX 1729/1 (Fig. 2G) exhibits only the central portion of the tooth crown, while most of the marginal areas are heavily damaged or missing. The occlusal ornamentation consists of concentric thin ridges, which are surrounded by concentric fine wrinkles and granules.

*Remarks*. NMNHU-P EX 1729/1 is too fragmentary to establish its position within the dentition confidently.

***Ptychodus polygyrus*** Agassiz, 1835

(Fig. 2E, 2H,H^I^)

For synonyms, see Amadori et al. (2020a: p. 7).

*Diagnosis*. See Amadori et al. (2020a: p. 8).

*Referred material*. Three teeth, NMNHU-P PI 2345, NMNHU-P PI 2346, PIMUZ A/I PIMUZ A/I 5234, all from Malyn.

*Description*. NMNHU-P PI 2346 (Fig. 2H,H^I^) and NMNHU-P PI 2345 have asymmetrical but almost rectangular tooth crowns. The crown of NMNHU-P PI 2346 has a straight edge on the right side, while the left one is tilted posteriorly (see Fig. 2H). In NMNHU-P PI 2345, the straight crown margin is on the left side of the crown. Six transversal ridges cross the occlusal surface of NMNHU-P PI 2346, reaching the lateral crown edges (see Fig. 2H). NMNHU-P PI 2345 has eleven occlusal ridges exhibiting an identical pattern. In both specimens, the ridges are slightly abraded. In posterior view (Fig. 2H^I^), the occlusal surface of NMNHU-P PI 2346 is flat, and the root is thicker than the crown. The antero-posterior sulcus in the root of NMNHU-P PI 2346 is almost absent (see Fig. 2H^I^). The crown of NMNHU-P PI 2345 is slightly raised centrally, while its root is missing. PIMUZ A/I 5234 displays 11 transversal ridges that do not reach the lateral edges of the crown. The ridges curve anteriorly at their ends (see Fig. 2E). Its lateral tooth margins are broken, although coarse granulations are recognizable on the marginal area. In PIMUZ A/I 5234, the crown is slightly bulgy in its central part, while the root is missing.

*Remarks*. Both NMNHU-P Pi 2346 (Fig. 2H^I^) and NMNHU-P PI 2345 are identified here as lateral teeth based on their asymmetrical crowns. The flat surface of NMNHU-P PI 2346 and the outline of its lateral crown edges suggest is original placement within the left hemiarch of the upper dentition. The bulgy occlusal surfaces of NMNHU-P PI 2345 and PIMUZ A/I 5234 suggest that they were positioned within the lower dental plate.

***Ptychodus*** sp. cf. ***P. anonymus*** Williston, 1900 (Fig. 2I,I^I^)

*Referred material*. Two teeth, NMNHU-P EX 1729/2 from Kaniv and NMNHU-P PI 1729/2 from Malyn.

*Description*. Both NMNHU-P EX 1729/2 (Fig. 2I,I^I^) and NMNHU-P PI 1729/2 are cusp fragments displaying a rounded and narrow apex. Six to seven thin ridges cross the entire cusp of NMNHU-P EX 1729/2 reaching its lateral surfaces (see Fig. 2I,I^I^). Only the anterior portion of the crown is preserved in NMNHU-P PI 1729/2. The ridges are slightly abraded on their cusp apices.

*Remarks*. The ornamentation pattern in NMNHU-P EX 1729/2 (Fig. 2I,I^I^) and NMNHU-P PI 1729/2 is similar to that described by Hamm (2020) for the cuspidate taxon *Ptychodus anonymus*. However, an unambiguous taxonomic identification is not possible for these specimens due to their fragmentary nature.

***Ptychodus*** sp. cf. *P*. ***mammillaris*** Agassiz, 1835

(Figs. 2J,J^I^, 2K,K^I^)

*Referred material*. Four teeth, NMNHU-P PI 1729/1, NMNHU-P PI 2348e50, all from Malyn.

*Description*. NMNHU-P PI 1729/1 (Fig. 2J,J^I^), NMNHU-P PI 2348 and NMNHU-P PI 2349 exhibit only the central portions of the crowns. The latter are characterized by low cusps. NMNHU-P PI 2350 (Fig. 2K,K^I^) also displays a flattened cusp with poorly preserved lateral areas. The apices of all four teeth are crossed by six to ten thin ridges.

*Remarks. Ptychodus mammillaris* is a cuspidate species characterized by a flat cusp apex and ridges that are limited to the center of the crown (see Hamm, 2020). Although the specimens presented here have low cusps, it is possible that they were flattened post-mortem due to abrasion as exemplified by the smoothened apex representing a taphonomic artefact. Due to the poor preservation and the high degree of occlusal abrasion of NMNHU-P PI 1729-1, NMNHU-P PI 2348, NMNHU-P PI 2349 and NMNHU-P PI 2348-50, their unambiguous attribution to *P. mammillaris* is impossible.

***Ptychodus*** sp. cf. ***P. marginalis*** Agassiz, 1839 (*sensu* Hamm, 2020) (Fig. 2C)

*Referred material*. A single tooth, NMNHU-P PI 2344, from Malyn.

*Description*. NMNHU-P PI 2344 (Fig. 2C) has a flat crown crossed by ten ridges, which merge at their lateral ends forming a concentrical pattern. The ridges are slightly abraded. Only a small part of the marginal area is preserved in NMNHU-P PI 2344 (Fig. 2C) exposing wrinkles on the occlusal surface with a possible concentrical pattern. *Remarks. Ptychodusmarginalis* isanun-cuspidatespeciescharacterized by concentric occlusal ornamentation both in the center and on the marginal areas of the crown (see also Amadori et al., 2020a). Although such pattern is well recognizable in the crown center of NMNHU-P PI 2344 (see Fig. 2C), the poor state of preservation of its marginal areas prevents a proper taxonomic identification of this specimen.

***Ptychodus*** sp. cf. ***P. polygyrus*** Agassiz, 1835

*Referred material*. Two teeth, NMNHU-P Pi 2343 and NMNHU-P PI 2347, from Malyn.

*Description*. Both teeth, NMNHU-P Pi 2343 and NMNHU-P Pi 2347 have rectangular tooth crowns with a shallow posterior sulcus and damaged and/or abraded external edges. Their occlusal surface exhibits seven to eight ridges curving anteriorly. The latter are markedly abraded especially at the center of the crown.

*Remarks*. Although some of the species-specific characters of *Ptychodus polygyrus* are recognizable in NMNHU-P PI 2343 and NMNHU-P PI 2347 (see also Amadori et al., 2020a), the damaged margins of their tooth crowns prevent an unambiguous species identification for these specimens.

***Ptychodus*** sp.

*Referred material*. Two teeth, NMNHU-P PI 1729/3-5 and NMNHU-P PI 2352, from Malyn.

*Description*. The specimens NMNHU-P PI 1729/3-5 are small-sized crown fragments, which are characterized by markedly abraded ridges. NMNHU-P PI 2352 exhibits a very small portion of the cusp crossed by abraded ridges.

*Remarks*. The ridges characterizing the surface of NMNHU-P PI 1729/3-5 and NMNHU-P PI 2352 suggest that they belong to the genus *Ptychodus*. A species level identification, however, is impossible due to the fragmentary nature of the specimens examined.

## 5 Discussion

### 5.1 Taxonomic composition

Despite the uncertain identification of some isolated teeth described herein, *Ptychodus* seemingly was well diversified in the shallow, epicontinental sea covering the Ukrainian Shield during the Cenomanian with both cuspidate and un-cuspidate taxa being present (see Table 1; see also Table S1 in online supplementary data). Most of the specimens documented here represent the oldest occurrences of *Ptychodus* in Ukraine (see Table 2; see also “Geological setting”, above). The taxonomic composition from the Cenomanian of Malyn is mostly consistent with that previously documented from other European localities of the same age (see Table 1; see also Amadori et al., 2022b).

**Fig. 3:**
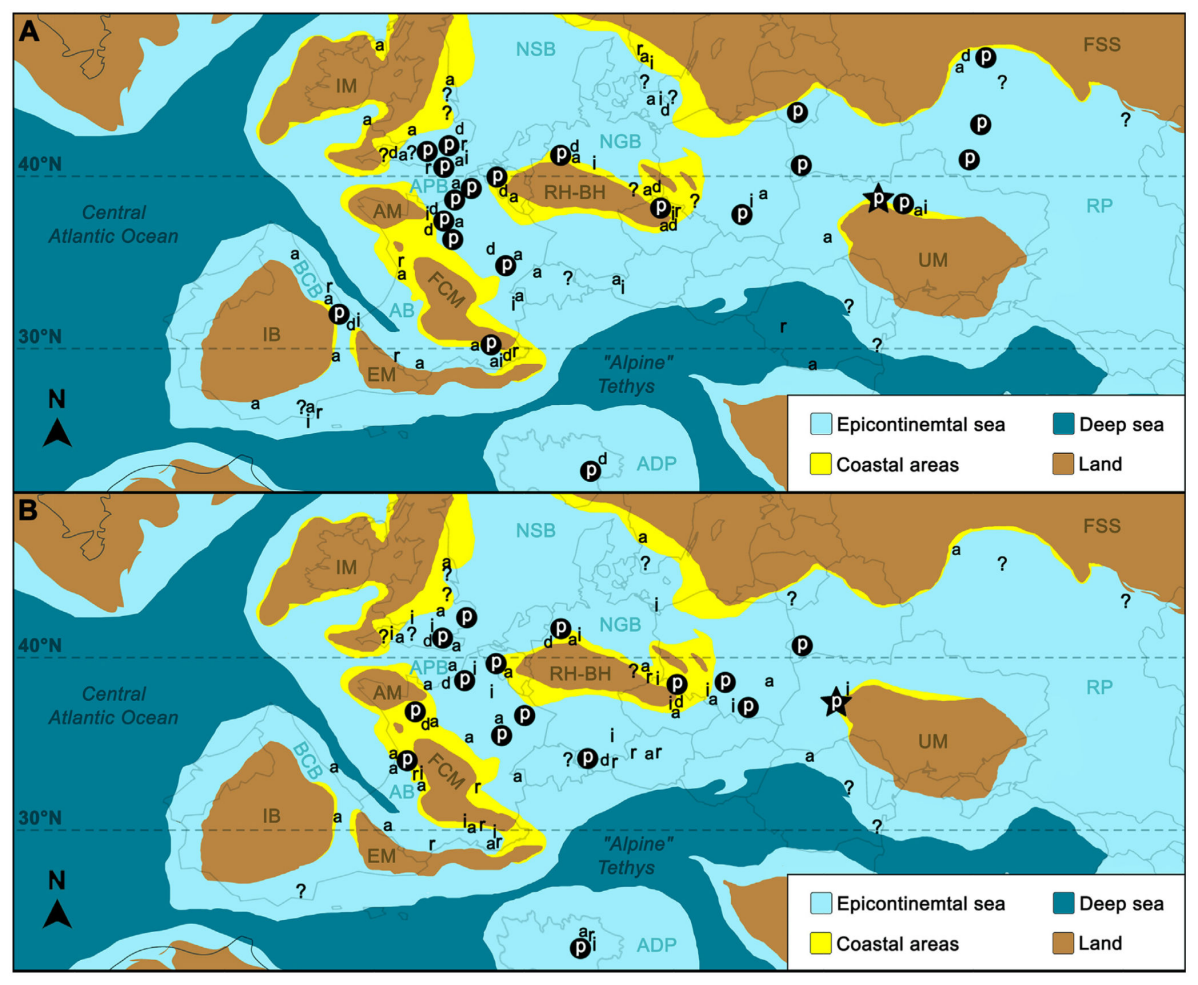
Late Cenomanian–early Turonian paleogeographic maps of Europe (modified after Amadori et al., 2022b; coastal seas based on Janetschke and Wilmsen, 2014; Le Callonnec et al., 2021) with Cenomanian (A, 100–94 Mya) and Turonian (B, 94–90 Mya) occurrences of *Ptychodus* (p) and its possible prey (a, ammonoids; d, decapods; i, inoceramids; r, rudists). See Table S2 (online supplementary data) for more details on mollusk occurrences. The black star indicates the occurrence of *Ptychodus* documented in the present paper; question marks indicate occurrences of *Ptychodus* of uncertain age. AB, Aquitaine Basin; ADP, Adria Platforms; APB, Anglo-Paris Basin; BCB, Basque-Cantabrian Basin; RH-BH, Rhenish-Bohemian High; IB, Iberia; IM, Irish Massif; FCM, French Massif Central; FSS, Fennoscandian Shield; NSB, North Sea Basin; RP, Russian Platform; UM, Ukrainian Massif.

The most diverse assemblages of *Ptychodus* during the early Late Cretaceous inhabited both coastal and outer shelf areas of the epicontinental seas at the north-western margin of the Peri-Tethys (see Amadori et al., 2022b). For instance, cuspidate and un-cuspidate species of *Ptychodus* were reported from the Cenomanian–Turonian of the Anglo-Paris Basin (see also Amadori et al., 2022b). In general, the “White Chalk” in southern England (northern Anglo-Paris Basin; APB in Fig. 3) was mostly deposited in an offshore environment between 100 and 600 m depth (epimesopelagic; see Scholle, 1974; Hancock, 1975). However, the margins of the Anglo-Paris Basin were characterized by mid- and inner-shelf environments during the Cenomanian, while deeper chalk facies are represented by Turonian deposits in the same area (e.g., Gale et al., 2000; Le Callonnec et al., 2021).

Cuspidate and un-cuspidate species of *Ptychodus* also inhabited coastal environments of the Münster Basin (Westphalia, north-western Germany; see Fig. 3) during the Cenomanian–Turonian. In the same area, only the un-cuspidate *P. latissimus* was documented from continental slope facies (outer shelf; see Diedrich, 2013). Although the taxonomic composition of assemblages of *Ptychodus* from various European localities may vary, cuspidate and un-cuspidate species are common in offshore and coastal deposits (see also Amadori et al., 2022b).

### 5.2 Prey availability, trophic strategies and feeding adaptations

Spatio-temporal variations in trophic ecology of sharks are very common (Newman et al., 2010; Wetherbee et al., 2012; Bizzarro et al., 2017). In fact, sharks can be very selective in their dietary range, but also switch to more opportunistic feeding strategies due to environmental changes (e.g., prey availability; Newman et al., 2010). Primary productivity and faunal diversity greatly increase approaching inshore areas, supporting diverse ecological niches (Nixon et al., 1986; Ross, 1986; Plumlee and Wells, 2016). Most coastal sharks are relatively stationary and opportunistically feed within the same area, whereas pelagic species actively reach possible hotspots of prey abundance (Bizzarro et al., 2017). Furthermore, durophagous sharks can access niches and food resources (e.g., hard-shelled items), which are not always available to most of their direct competitors through a set of morphological adaptations correlated with their feeding strategies (e.g., crushing dentitions; Wilga and Motta, 2000). However, opportunistic trophic strategies cannot be excluded even for durophagous specialists lacking pointed, cuspidate teeth (grasping dentition), as it occurs in some groups of extant batoids (Gilliam and Sullivan, 1993; Ebert and Bizzarro, 2007; López-García et al., 2012; Jacobsen and Bennett, 2013). For example, dental cusps are absent in the dentition of extant guitarfishes (Batomorphii, Elasmobranchii), such as *Rhina ancylostoma* and *Zapteryx exasperate* (see Herman et al., 1997; Berkovitz and Shellis, 2017). Nevertheless, their un-cuspidate dental plates do not prevent these batoids from occasionally consuming small teleost fishes in addition to their main prey (e.g., crustaceans; Purushottama et al., 2022; Reyes-Ramíre et al., 2022). In elasmobranchs, variation of the “niche breadth” (*sensu* Bazzi et al., 2021) is crucial for avoiding resource competition between predators with similar trophic adaptations, such as dental plates in batoids (see Bornatowski et al., 2014; Munroe et al., 2014; Lear et al., 2021). Adaptations and modifications affecting the functionality of the feeding apparatus undoubtedly have a great impact on the survival of individuals and can determine the success of particular evolutionary lineages (Schwenk, 2000; Bels and Herrel, 2019). Consequently, the study of trophic systems can increase our understanding of possible evolutionary trajectories for predatory groups (see Gans, 1994).

Primary productivity and, consequently, general nutrient availability in epicontinental seas increased greatly during most of the Cenomanian with the exception of the Oceanic Anoxic Event 2 (OAE2) interval at the Cenomanian–Turonian boundary (Pearce et al., 2009; Linnert et al., 2011). Furthermore, Mesozoic bivalves experienced the greatest genus-level diversification during the “mid-Cretaceous” (Albian-Turonian; Mondal and Harries, 2015). Although the overall ammonite diversity decreased during the Late Cretaceous (Yacobucci, 2015), this group of shelled cephalopods underwent a major Cenomanian–Turonian turnover, which could have directly affected hard-shelled specialist feeding during that time (Monnet, 2009). In addition, the sea level rise during the Cenomanian–Turonian triggered diversification of endemic ammonites within epicontinental seaways (Courville, 2007). Therefore, Cenomanian epicontinental environments likely supported highly abundant and diverse faunas of shelled mollusks and crustaceans (both primary consumers; see Cortés, 1999). Consequently, such highly productive habitats likely favored diverse predatory marine assemblages including abundant co-occurring species of *Ptychodus* (see also Fig. 3).

Preliminary studies on global occurrences of *Ptychodus* indicate that the early Late Cretaceous was probably the most successful time for these durophagous predators with a first significant diversification during the Cenomanian (e.g., Hamm, 2020; Amadori, 2022). In Europe, species of *Ptychodus* were widespread in epicontinental seas from the marginal areas of the Basque-Cantabrian Basin (BCB in Fig. 3) to the Russian Platform (RP in Fig. 3), showing a great diversity with at least ten species during most of the Late Cretaceous (see also Amadori et al., 2022b). In the Cenomanian, cuspidate and un-cuspidate taxa of *Ptychodus* shared same habitats (epicontinental environments; Amadori et al., 2022b) and were distributed across most of Europe, inhabiting both coastal and offshore areas (see above). This would suggest niche partitioning possibly related to prey adaptation to reduce inter-species competition. Indeed, the occurrence of numerous species of *Ptychodus* with different tooth morphologies (e.g., flat crowns and knob-like, rounded, or sharp-tipped cusps) already in the Cenomanian (see Amadori et al., 2022b) could have been the result of a strong competition for food resources within this group of hard-prey specialists (“Red Queen hypothesis”; see van Valen, 1973; Benton, 2009). For instance, cuspidate forms were likely capable of hunting more elusive soft-body prey, such as squids or small fishes, catching them with their grasping dentitions (e.g., Shimada, 2012; Amadori et al., 2022a). Moreover, at least some species of *Ptychodus* were probably fast swimmers (see Hamm, 2010) able to cross open marine environments. Nevertheless, a wide distribution of *Ptychodus* species in Cenomanian epicontinental seas suggests that this durophagous predator was a pelagic dweller rather than strictly associated with coastal environments. *Ptychodus* species thus could have sought new hotspots of prey abundance across Europe and found there a “breeding ground” for its subsequent maximum success in terms of species diversity and geographical distribution during the Turonian-Coniacian (e.g., Hamm, 2020; Amadori, 2022). However, sharks are highly susceptible to climatic and environmental fluctuations (e.g., Bernal et al., 2012; Schlaff et al., 2014; Munroe et al., 2014; Reinero et al., 2022). Therefore, abiotic factors during the early Late Cretaceous, such as marine transgressions and climate warming of surface waters (e.g., Pearce et al., 2009; Linnert et al., 2011; Jouve et al., 2017), could have also driven the diversification of *Ptychodus* (“Court Jester hypothesis”; Barnosky, 2001; Benton, 2009). A combined effect of “Court Jester” and “Red Queen” could have thus influenced the evolutionary path of this group of durophagous sharks (see also Benton, 2009; Condamine et al., 2019; Amadori, 2022). However, future studies and tests are needed to evaluate and verify the hypothesis proposed here.

## 6 Conclusions

The assemblage of *Ptychodus* species from the lower Upper Cretaceous of Ukraine documented here is relatively diverse with five identified species, even though each of them is represented by only very few specimens. Both cuspidate (*P. altior*) and un-cuspidate (*P. decurrens, P. latissimus, P. marginalis* and *P. polygyrus*) species inhabited coastal epicontinental seas at the north-western edge of the Ukrainian Massif. The Cenomanian occurrence of two other species (*P. anonymus* and *P. mammillaris*) remains speculative and their presence in this part of the European Peri-Tethys needs to be confirmed in future studies when more abundant and better-preserved material will be recovered.

The abundant and diverse faunas of shelled macroinvertebrates (e.g., bivalves, ammonites, and decapod crustaceans) co-occurring in Cenomanian epicontinental environments probably favored the initial diversification of *Ptychodus* with various species adapted to hunt and feed on different prey. The presence of several species of *Ptychodus* in various Cenomanian inshore and offshore areas across the European epicontinental seas allows hypothesizing niche partitioning between cuspidate and un-cuspidate species. The ultimate diversification of *Ptychodus* during the Cenomanian–Turonian thus was partially supported by niche differentiation to avoid an escalating competition within the genus. Assuming a pelagic lifestyle for *Ptychodus*, the active seeking of new suitable environments characterized by prey abundance could have been an additional countermeasure against competition within the genus. However, further in-depth studies on the Late Cretaceous diversity and dispersal patterns of *Ptychodus* correlated with possible fluctuations in biotic and abiotic environmental factors are mandatory for identifying local niche partitioning and/or spatial separation between cuspidate and un-cuspidate taxa.

## Supplementary Material

**Appendix A. Supplementary data**

Supplementary data to this article can be found online at https://doi.org/10.1016/j.cretres.2023.105659.

Table 1

Table 2

## Figures and Tables

**Table 1:** Occurrences of *Ptychodus* from the Cenomanian–Turonian of Ukraine (UKR) compared with those previously reported from Europe (EU; data on European occurrences from Amadori et al., 2022b: tables S3, supplemental data). Asterisk indicates occurrences documented in the present study. Dubious occurrences are excluded. C, cuspidate taxon; UC, un-cuspidate taxon.

Species	Cen (UKR)	Tur (UKR)	Cen (EU)	Tur (EU)
*P. altior* (C)	✓*			**✓**
*P. anonymus* (C)			**✓**	**✓**
*P. decurrens* (UC)			**✓**	**✓**
*P. latissimus* (UC)		✓*	**✓**	**✓**
*P. mammillaris* (C)		**✓**	**✓**	**✓**
*P. marginalis* (UC)	✓*		**✓**	
*P. mediterraneus* (UC)				**✓**
*P. polygyrus* (UC)	✓*		**✓**	**✓**

**Table 2:** Taxonomical composition of *Ptychodus* from the Upper Cretaceous of the territory of modern Ukraine (Asterisk indicates occurrences documented in the present study). C, cuspidate taxon; UC, un-cuspidate taxon.

Species	Cenomanian			Turonian			Coniacian
	Malyn	Kaniv		Kremenets	Zdolbuniv		Bukivna
*P. altior* (C)	✓*						
*P. anonymus* (C)	?*	?*					
*P. decurrens* (UC)	✓*						
*P. mammillaris* (C)	?*			**✓**	**✓**		
*P. marginalis* (UC)	✓*	✓*					
*P. latissimus* (UC)				✓*			
*P. polygyrus* (UC)	✓*						**✓**

## Data Availability

Data used in this study are available in the Appendix A (see online Supplementary material).
