# Supplementary material for: A diverse assemblage of Ptychodus species (Elasmobranchii: Ptychodontidae) from the Upper Cretaceous of Ukraine, with comments on possible diversification drivers during the Cenomanian
Source: Cretac Res. Author manuscript; Available in PMC 2024 May 26. (PMC7615990; doi:10.1016/j.cretres.2023.105659)
Supplement: Table 1 [file EMS196223-supplement-Table_1.docx]

| **Catalogue N°** | **Specimen** | **Provenance** | **Identification** | **WT** |
| --- | --- | --- | --- | --- |
| NMNHU-G 391/15 | Broken tooth crown | Kremenets | *P. latissimus* (UC) |  |
| NMNHU-P Ex 1729/1 | Broken tooth crown | Kaniv | *P. marginalis* (UC) | ✓ |
| NMNHU-P Ex 1729/2 | Dental cusp apex | Kaniv | *P.* cf. *anonymus* (C) | ✓ |
| NMNHU-P Pi 1729/1 | Tooth crown | Malyn | *P.* sp. cf. *P.* *mammillaris* (C) | ✓ |
| NMNHU-P Pi 1729/2 | Broken tooth crown | Malyn | *P.* sp. cf. *P.* *anonymus* (C) | ✓ |
| NMNHU-P Pi 1729/3 | Tooth fragment | Malyn | *Ptychodus* sp. | ? |
| NMNHU-P Pi 1729/4 | Tooth fragment | Malyn | *Ptychodus* sp. | ✓ |
| NMNHU-P Pi 1729/5 | Tooth fragment | Malyn | *Ptychodus* sp. | ? |
| NMNHU-P Pi 2342 | Tooth crown | Malyn | *P. decurrens* (UC) | ✓ |
| NMNHU-P Pi 2343 | Tooth crown | Malyn | *P.* sp. cf. *P.* *polygyrus* (UC) | ✓ |
| NMNHU-P Pi 2344 | Broken tooth crown | Malyn | *P.* sp. cf. *P.*  *marginalis* (UC) | ✓ |
| NMNHU-P Pi 2345 | Tooth crown | Malyn | *P. polygyrus* (UC) | ✓ |
| NMNHU-P Pi 2346 | Tooth crown | Malyn | *P. polygyrus* (UC) |  |
| NMNHU-P Pi 2347 | Tooth crown | Malyn | *P.* sp. cf. *P.* *polygyrus* (UC) | ✓ |
| NMNHU-P Pi 2348 | Dental cusp apex | Malyn | *P.* sp. cf. *P.* *mammillaris* (C) | ✓ |
| NMNHU-P Pi 2349 | Broken tooth crown | Malyn | *P.* sp. cf. *P.* *mammillaris* (C) | ✓ |
| NMNHU-P Pi 2350 | Tooth crown | Malyn | *P.* sp. cf. *P.*  *mammillaris* (C) | ✓ |
| NMNHU-P Pi 2351 | Tooth crown | Malyn | *P. altior* (C) | ✓ |
| NMNHU-P Pi 2352 | Tooth fragment | Malyn | *Ptychodus* sp. | ✓ |
| PIMUZ A/I 5234 | Tooth crown | Malyn | *P. polygyrus* (UC) |  |
| PIMUZ A/I 5235 | Tooth crown | Malyn | *P. decurrens* (UC) | ✓ |
| PIMUZ A/I 5236 | Tooth crown | Malyn | *P. decurrens* (UC) | ✓ |
| PIMUZ A/I 5237 | Tooth crown | Malyn | *P. decurrens* (UC) | ✓ |
