# Supplementary material for: A diverse assemblage of Ptychodus species (Elasmobranchii: Ptychodontidae) from the Upper Cretaceous of Ukraine, with comments on possible diversification drivers during the Cenomanian
Source: Cretac Res. Author manuscript; Available in PMC 2024 May 26. (PMC7615990; doi:10.1016/j.cretres.2023.105659)
Supplement: Table 2 [file EMS196223-supplement-Table_2.docx]

| **Ammonites** |
| --- |
| Amédro, F., Matrion, B., & Robaszynski, F. (2006). Romaniceras (Yubariceras) Ornatissimum (Stolickza, 1864): Une Ammonite Rare Dans Les Craies Du Turonien Moyen De L'aube (France) Romaniceras (Yubariceras). Bulletin d'information des géologues du bassin de Paris, 43(4), 17-25. |
| Barroso-Barcenilla, F. (2004). Acanthoceratidae y zonación de ammonites del Cenomaniense superior y del Turoniense inferior en el área de Puentedey, Cuenca Vasco-Cantábrica, España Acanthoceratidae and ammonite zonation of the upper Cenomanian and the lower Turonian in the Puentedey area. Coloquios de Paleontología, 54, 83-114. |
| Barroso-Barcenilla, F., & Goy, A. (2010). The ammonite genus Vascoceras Choffat, 1898 (family Vascoceratidae Douvillé, 1912) in the Iberian Trough, Spain. Palaeontographica Abteilung A, 199-235. |
| Bergström, J., Christensen, W. K., Johansson, C., & Norling, E. (1973). An extension of Upper Cretaceous rocks to the Swedish west coast at Särdal. Bulletin of the Geological Society of Denmark, 22(02), 83-154. |
| Berrocal-Casero, M., Barroso Barcenilla, F., Callapez, P., García Joral, F., & Segura, M. (2013). Bioestratigrafía de macrofósiles del Cenomaniense Superior-Turoniense Inferior en el área de Satamera y Riofrío del Llano (Guadalajara, España). Revista de la Sociedad Geológica de España, 26(2), 85-106. |
| Bert, D., Perès, V., & Marchand, D. (2006). Nouvelles données stratigraphiques sur le Turonien moyen (Crétacé supérieur) du versant septentrionale du seuil du Poitou (Centre-Ouest de la France): description de Pseudotissotia faustinleybachae sp. nov. et evolution des Pseudotissotinae Hyatt, 1903 (Ammonoidea) oust-européens. In Annales du Muséum d’Histoire naturelle de Nice (Vol. 21, pp. 297-317). |
| Birkelund, T. (1957). Upper Cretaceous belemnites from Denmark. I kommission hos Munksgaard, 9(1), 1-69. |
| Delamette, M. J., Charollais, J. J., Decrouez, D., & Caron, M. (1997). Les grès verts helvétiques (Aptien moyen-Albien supérieur) de Haute-Savoie, Valais et Vaud (Alpes occidentales Franco-Suisses): Analyse stratigraphique et inventaire paléontologique. Sectiondes Sciences de la Terre, 23, 1-400. |
| Diebold, F., Bengtson, P., Lees, J. A., & Walaszczyk, I. (2010). Ammonite, inoceramid and nannofossil biostratigraphy across the Turonian–Coniacian boundary in the Aquitaine and Vocontian basins (France) and Diego Basin (Madagascar). In Abstract Volume of the 8th International Symposium, Cephalopods–Present and Past (Dijon–France 2010) (p. 35). |
| Gale, A. S., Kennedy, W. J., Burnett, J. A., Caron, M., & Kidd, B. E. (1996). The Late Albian to Early Cenomanian succession at Mont Risou near Rosans (Drôme, SE France): an integrated study (ammonites, inoceramids, planktonic foraminifera, nannofossils, oxygen and carbon isotopes). Cretaceous Research, 17(5), 515-606. |
| Gale, A. S., Simms, M. J., & Kennedy, W. J. (2018). Stratigraphy and ammonite faunas of the Cenomanian rocks of Northern Ireland, UK. Cretaceous Research, 87, 102-119. |
| Gallemi, J., Martinez, R., & Pons, J. M. (1983). Coniacian-Maastrichtian of the Tremp area (south central Pyrenees). Newsletters on Stratigraphy, 1-17. |
| Hardenbol, J., Caron, M., Amedro, F., Dupuis, C., & Robaszynski, F. (1993). The Cenomanian-Turonian boundary in central Tunisia in the context of a sequence-stratigraphic interpretation. Cretaceous Research, 14(4-5), 449-454. |
| Hauschke, N. (1994). Lepadomorphe Cirripedier (Crustacea, Thoracica) aus dem höchsten Cenoman des nördlichen Westfalen (Nordwestdeutschland), mit Bemerkungen zur Verbreitung, Palökologie und Taphonomie der Stramentiden. Landschaftsverband Westfalen-Lippe. |
| Jaccard, A. (1869). Description géologique du Jura Vaudois et Neuchatelois. Matériaux pour la Cart. géologique la. Suisse, 6, 1-340. |
| Keller, S. (1982). Die Oberkreide der Sack-Mulde bei Alfeld-(Cenoman-Unter-Coniac)-Lithologie, Biostratigraphie-und Inoceramen. |
| Kennedy, W. J. (1970). A correlation of the uppermost Albian and the Cenomanian of south-west England. Proceedings of the Geologists' Association, 81(4), 613-676. |
| Kennedy, W. J. (1994). Lower Turonian ammonites from Gard (France). Palaeopelagos special publication, 1, 255-275. |
| Kennedy, W. J., & Gale, A. S. (2015). Late Turonian ammonites from Haute-Normandie, France. Acta Geologica Polonica, 65(4). |
| Kennedy, W. J., & Gale, A. S. (2016). Turonian ammonites from northwestern Aquitaine, France. Cretaceous Research, 58, 265-296. |
| Kennedy, W. J., & Jolkicev, N. (2004). Middle Cenomanian ammonites from the type section of the Sanandinovo Formation of northern Bulgaria. Acta Geologica Polonica, 54(3), 369-380. |
| Kennedy, W. J., Bilotte, M., & Melchior, P. (1995). Ammonite faunas, biostratigraphy and sequence stratigraphy of the Coniacian-Santonian of the Corbières (NE Pyrénées). Bulletin des Centres de Recherches Exploration-Production Elf Aquitaine, 19(2), 377-499. |
| Kennedy, W. J., Amédro, F., Robaszynski, F., & Jagt, J. W. (2011). Ammonite faunas from condensed Cenomanian-Turonian sections (‘Tourtias’) in southern Belgium and northern France. Netherlands Journal of Geosciences, 90(2-3), 209-238. |
| Kin, A., & Niedźwiedzki, R. (2012). First record of the puzosiine ammonite genus Pachydesmoceras from the Middle and Upper Turonian of Poland. Cretaceous Research, 33(1), 15-20. |
| Košťák, M., Čech, S., Uličný, D., Sklenář, J., Ekrt, B., & Mazuch, M. (2018). Ammonites, inoceramids and stable carbon isotopes of the Cenomanian–Turonian OAE2 interval in central Europe: Pecínov quarry, Bohemian Cretaceous Basin (Czech Republic). Cretaceous Research, 87, 150-173. |
| Linares, A., & Cremades, J. (1988). Graysonites (Mantelliceratinae Ammonitina) from the LowermostCenomanian of Betic Cordillera (Spain). Geobios, 21(3), 307-317. |
| Neraudeau, D., & Moreau, P. (1996). Paléoécologie et paléobiogéographie des faunes d'échinides du Cénomanien nord-aquitain (Charente-Maritime, France). Geobios, 22(3), 293-324. |
| Paul, C. R. C., Mitchell, S. F., Marshall, J. D., Leafy, P. N., Gale, A. S., Duane, A. M., & Ditchfield, P. W. (1994). Palaeoceanographic events in the middle Cenomanian of Northwest Europe. Cretaceous Research, 15(6), 707-738. |
| Renz. O. (1976). Ein grosser Pachydesmoceras (Ammoniodea) aus dem Unteren Cénomanien des Schweizer Juras. Eclogae Geologicae Helvetiae, 69:753-763 |
| Sklenář, J., & Simon, E. (2009). Brachiopod Gyrosoria Cooper, 1973–a comparative palaeoecological, stratigraphical and taxonomical study. Bulletin of Geosciences, 84(3), 437-464. |
| Smettan, K. (1997). Bivalven, Gastropoden und Serpuliden aus den Branderfleckschichten (Cenoman) der Fahrenbergmulde (Nördliche Kalkalpen, Bayern): Taxonomie und Palökologie. Zitteliana, 21, 99-158. |
| Storc, R., & Zitt, J. (2008). Late Turonian ophiuroids (Echinodermata) from the Bohemian Cretaceous Basin, Czech Republic. Bull Geosci, 83, 123-140. |
| Summesberger, H. (1992). Ammoniten aus dem Turon (Oberkreide) der Nördlichen Kalkalpen (Österreich). Annalen des Naturhistorischen Museums in Wien. Serie A für Mineralogie und Petrographie, Geologie und Paläontologie, Anthropologie und Prähistorie, 94, 103-133. |
| Świerczewska-Gładysz, E., & Jurkowska, A. (2013). Occurrence and paleoecological significance of lyssacinosid sponges in the Upper Cretaceous deposits of southern Poland. Facies, 59, 763-777. |
| Szasz, L. (1984). Lower Turonian ammonite assemblage in the Maramures Mountains (East Carpathians-Romania). Dări de seamă ale sedintelor. Institutul de geologie si geofizică. 3. Paleontologie, 70(3), 117-134. |
| Trevisani, E., & Cestari, R., (2007). Upper Cretaceous bivalves from basinal highs (Venetian Prealps, northern Italy). In: Scott, R.W. (Ed.), Cretaceous Rudists and Carbonate Platforms: Environmental Feedback, vol. 87. SEPM Special Publication, pp. 71-80. |
| Valette, A. (1914). Les Ophiures de la Craie des Environs de Sens, Bulletin de la Société des Sciences Historiques et Naturelles de l'Yonne, 68(18), 125-150. |
| Vullo, R., Bernárdez, E., & Buscalioni, A. D. (2009). Vertebrates from the middle?–late Cenomanian La Cabaña Formation (Asturias, northern Spain): Palaeoenvironmental and palaeobiogeographic implications. Palaeogeography, Palaeoclimatology, Palaeoecology, 276(1-4), 120-129. |
| Wilmsen, M., & Nagm, E. (2013). Upper Cenomanian-Lower Turonian ammonoids from the Saxonian Cretaceous (lower Elbtal Group, Saxony, Germany). Bulletin of Geosciences, 88(3), 647-674. |
| Wilmsen, M., & Nagm, E. (2014). Kreide-Fossilien in Sachsen, Teil 1, 7. Ammoniten. Geologica Saxonica, 60, 201-240 |
| Woods, M. A. (2002). The macrofossil biostratigraphy of the Turonian and Coniacian (Upper Cretaceous, Chalk Group) of southeast Devon. Proceedings of the Geologists' Association, 113(4), 333-344. |
| Wright, C. W. (1979). The ammonites of the English Chalk Rock (Upper Turonian). Bulletin of the British Museum of Natural History (Geology), 31, 281-332 |
| Žítt, J., Vodrážka, R., Hradecká, L., Svobodová, M., & Zágoršek, K. (2006). Late Cretaceous environments and communities as recorded at Chrtníky (Bohemian Cretaceous Basin, Czech Republic). Bulletin of Geosciences, 81(1), 43-79. |
| **Decapods** |
| Bert, D., Perès, V., & Marchand, D. (2006). Nouvelles données stratigraphiques sur le Turonien moyen (Crétacé supérieur) du versant septentrionale du seuil du Poitou (Centre-Ouest de la France): description de Pseudotissotia faustinleybachae sp. nov. et evolution des Pseudotissotinae Hyatt, 1903 (Ammonoidea) oust-européens. In Annales du Muséum d’Histoire naturelle de Nice (Vol. 21, pp. 297-317). |
| Breton, G. (2006). Un cas de paléocoprophagie chez un crabe du Cénomanien du Mans (Collection Boutillier, Université de Caen). Bulletin de la Société linnéenne de Normandie, 119, 41-43. |
| Breton, G. (2008). Une carapace de crabe préservée dans le moule interne d’un gastropode du Cénomanien de Basse-Normandie (France). L’écho des falaises, 12, 51-57. |
| Dhondt, A. V., & Dieni, I. (1993). Non-rudistid Bivalves from Late Cretaceous Rudist Limestones of NE Italy:(Col Dei Schiosi and Lago Di S. Croce Areas). Memorie di Scienze Geologiche, 45, 165-241. |
| Kelly, S. R. A., & Bromley, R. G. (1984). Ichnological, nomenclature of clavate borings Palaeontology 27, 793-807. |
| Jagt, J. W., Van Bakel, B. W., & Fraaije, R. H. (2007). Palaeodromites crypticus, early-middle Cenomanian dynomenid crab (Crustacea, Decapoda) from southern Belgium. bulletin de l'institut royal des sciences naturelles de belgique sciences de la terre, 77, 77-82. |
| Jarvis, I., Gale, A., & Clayton, C. (1982). Litho-and biostratigraphical observations on the type sections of the Craie de Villedieu Formation (Upper Cretaceous, western France). Newsletters on Stratigraphy, 64-82. |
| Jefferies, R. P. S. (1962). The palaeoecology of the Actinocamax plenus subzone (lowest Turonian) in the Anglo-Paris Basin. Palaeontology, 4(4), 609-647. |
| M'Coy, F. (1849). XLI.—On the classification of some British fossil Crustacea, with notices of new forms in the University Collection at Cambridge. Annals and Magazine of natural History, 4(24), 392-414. |
| Ossó, À. (2016). Eogeryon elegius n. gen. and n. sp.(Decapoda: Eubrachyura: Portunoidea), one of the oldest modern crabs from late Cenomanian of the Iberian Peninsula. Boletín de la Sociedad Geológica Mexicana, 68(2), 231-246. |
| Owen, E. F. (1988). Cenomanian brachiopods from the Lower Challe of Britain and northern Europe. Bulletin of the British Museum, Natural History. Geology, 44(2), 65-175. |
| Ruiz de Gaona, M. (1943). Nota sobre crustáceos decápodos de la cantera del Monte Orobe (Alsasua). Boletín de la Real Sociedad Española de Historia Natural. Sección Geológica, (41), 425-433. |
| Sanders, D. (1996). The Upper Cretaceous near Maurach (Tyrol, Austria). Geol. Paläontol. Mitt. Innsbruck, 21, 123-151. |
| Schlüter, C. A. J. (1862). Die Macruren Decapoden der Senon-und Cenoman-Bildungen Westphalens. |
| Van Bakel, B. W., Mychko, E. V., Spiridonov, A., Jagt, J. W., & Fraaije, R. H. (2021). New Cretaceous crabs (Crustacea, Brachyura) from Moscow Oblast and Dagestan (Russia): patterns in phylogeny and morphospace of the oldest eubrachyurans (Dorippoidea). Cretaceous Research, 119, 104675. |
| Van Straelen, V. (1936). Crustacés Décapodes nouveaux ou peu connus de l’époque Crétacique. *Bulletin du Musée royal d’Histoire naturelle de Belgique*, *12*(45), 1-50. |
| Von der Marck, W., & Schlüter, C. (1868). Neue Fische und Krebse aus der Kreide von Westphalen. Palaeontographica, 15(6), 269-305. |
| Veselská, M. K., Kočí, T., & Kubajko, M. (2014). Dynomenid crabs (Decapoda, Brachyura) and stalked barnacles (Cirripedia, Scalpelliformes) from upper Cenomanian-lower Turonian nearshore, shallow-water strata in the Bohemian Cretaceous Basin, Czech Republic. Scripta Geologica, 147, 49-81. |
| Žítt, J., Vodrážka, R., Hradecká, L., Svobodová, M., & Zágoršek, K. (2006). Late Cretaceous environments and communities as recorded at Chrtníky (Bohemian Cretaceous Basin, Czech Republic). Bulletin of Geosciences, 81(1), 43-79. |
| **Inoceramids** |
| Bergström, J., Christensen, W. K., Johansson, C., & Norling, E. (1973). An extension of Upper Cretaceous rocks to the Swedish west coast at Särdal. Bulletin of the Geological Society of Denmark, 22(02), 83-154. |
| Birkelund, T. (1957). Upper Cretaceous belemnites from Denmark. I kommission hos Munksgaard, 9(1), 1-69. |
| G. Breton. 1992. Les Goniasteridae (Asteroidea, Echinodermata) jurassiques et crétacés de France. Taphonomie, systématique, biostratigraphie, paléobiogéographie, évolution. Bulletin trimestriel de la Société géologique de Normandie et des Amis du Muséum du Havre, numéro hors série, 78:1-590 |
| Breton, G. (2008). Une carapace de crabe préservée dans le moule interne d’un gastropode du Cénomanien de Basse-Normandie (France). L’écho des falaises, 12, 51-57. |
| Cieslinski S. (1956). Stratigraphy and tectonics of the Cretaceous between Dobromierz, Józefów and Przedbórz on the Pilica (Middle Poland). Biuletyn Instytutu Geologicznego, 113, 139-194. |
| Delamette, M. J., Charollais, J. J., Decrouez, D., & Caron, M. (1997). Les grès verts helvétiques (Aptien moyen-Albien supérieur) de Haute-Savoie, Valais et Vaud (Alpes occidentales Franco-Suisses): Analyse stratigraphique et inventaire paléontologique. Sectiondes Sciences de la Terre, 23, 1-400. |
| Diebold, F., Bengtson, P., Lees, J. A., & Walaszczyk, I. (2010). Ammonite, inoceramid and nannofossil biostratigraphy across the Turonian–Coniacian boundary in the Aquitaine and Vocontian basins (France) and Diego Basin (Madagascar). In Abstract Volume of the 8th International Symposium, Cephalopods–Present and Past (Dijon–France 2010) (p. 35). |
| El-Shazly, S., Košťák, M., Abdel-Gawad, G., Kloukčová, B., Saber, S. G., Salama, Y. F., Mazuch, M., & Žák, K. (2011). Carbon and oxygen stable isotopes of selected Cenomanian and Turonian rudists from Egypt and Czech Republic, and a note on changes in rudist diversity. Bulletin of Geosciences, 86(2), 209-226. |
| Gale, A. S., Kennedy, W. J., Burnett, J. A., Caron, M. I. C. H. E. L., & Kidd, B. E. (1996). The Late Albian to Early Cenomanian succession at Mont Risou near Rosans (Drôme, SE France): an integrated study (ammonites, inoceramids, planktonic foraminifera, nannofossils, oxygen and carbon isotopes). Cretaceous Research, 17(5), 515-606. |
| Gallemı, J., López, G., Martınez, R., Muñoz, J., & Pons, J. M. (1997). Albian-Cenomanian and Campanian-Maastrichtian biostratigraphy of southeast Spain. Cretaceous Research, 18(3), 355-372. |
| Huxley, T. H. (1867). IV.—On Acanthopholis Horridus, a New Reptile from the Chalk-marl. Geological Magazine, 4(32), 65-67. |
| Jaccard, A. (1869). Description géologique du Jura Vaudois et Neuchatelois. Matériaux pour la Cart. géologique la. Suisse, 6, 1-340. |
| Jefferies, R. P. S. (1962). The palaeoecology of the Actinocamax plenus subzone (lowest Turonian) in the Anglo-Paris Basin. Palaeontology, 4(4), 609-647. |
| Keller, S. (1982). Die Oberkreide der Sack-Mulde bei Alfeld-(Cenoman-Unter-Coniac)-Lithologie, Biostratigraphie-und Inoceramen. |
| Kennedy, W. J. (1970). A correlation of the uppermost Albian and the Cenomanian of south-west England. Proceedings of the Geologists' Association, 81(4), 613-676. |
| Kennedy, W. J. (1994). Cenomanian ammonites from Cassis, Bouches-du-Rhône, France. Palaeopelagos Special Publication, 1, 209-254. |
| Kostak, M., Vodrazka, R., Frank, J., MazuCh, M., & MaReK, J. (2010). Late Cretaceous nautilid beaks from near-shore/shallow water deposits of the Bohemian Cretaceous Basin (Czech Republic). Acta Geologica Polonica, 60(3), 417-428. |
| Küchler, T. (1998). Upper Cretaceous of the Barranca (Navarra, northern Spain); integrated 1itho-, bioand event stratigraphy. Part I: Cenomanian through Santonian. Acta Geologica Polonica, 48(2), 157-236. |
| Lamolda, M. A., Gorostidi, A., Martı́nez, R., López, G., & Peryt, D. (1997). Fossil occurrences in the Upper Cenomanian-Lower Turonian at Ganuza, northern Spain: an approach to Cenomanian/Turonian boundary chronostratigraphy. Cretaceous Research, 18(3), 331-353. |
| Lehmann, J. (1999). Integrated stratigraphy and palaeoenvironment of the Cenomanian-Lower Turonian (Upper Cretaceous) of northern Westphalia, north Germany. Facies, 40, 25-69. |
| Malartre, F. (1994). Stratigraphie séquentielle du Crétacé supérieur du bassin vocontien occidental (Sud-Est, France). Comparaison avec d'autres bassins. Travaux et Documents des Laboratoires de Géologie de Lyon, 131(1), 3-219. |
| Paul, C. R. C., Mitchell, S. F., Marshall, J. D., Leafy, P. N., Gale, A. S., Duane, A. M., & Ditchfield, P. W. (1994). Palaeoceanographic events in the middle Cenomanian of Northwest Europe. Cretaceous Research, 15(6), 707-738. |
| Reich, M., & Wiese, F. (2010). Apodid sea cucumbers (Echinodermata: Holothuroidea) from the Upper Turonian of the Isle of Wolin, NW Poland. Cretaceous Research, 31, 350-363. |
| Salamon, M. A., Gorzelak, P., Borszcz, T., Gajerski, A., & Kaźmierczak, J. (2009). A crinoid concentration Lagerstätte in the Turonian (Late Cretaceous) Conulus Bed (Miechów-Wolbrom area, Poland). Geobios, 42(3), 351-357. |
| Schröder, H. (1885). Saurierreste aus der baltischen Obe-ren Kreide.Jahrbuch der Ko ̈niglich Preussischen Geo-logischen Landesanstalt und Bergakademie zu Berlin,1884, 293-333 |
| Smettan, K. (1997). Bivalven, Gastropoden und Serpuliden aus den Branderfleckschichten (Cenoman) der Fahrenbergmulde (Nördliche Kalkalpen, Bayern): Taxonomie und Palökologie. Zitteliana, 21, 99-158. |
| Sornay, J. (1983). Etude biostratigraphique des faunes d'Inocérames du Sénonien français. Géologie Méditerranéenne, 10(3), 193-198. |
| Soukup, J., (1938). Der erste Fund eines fossilen Seesterns in den Quadersandsteinen des Oberturons bei Jičin. Vierteljahrsschrift für Geologie und Erdkunde der Sudetenländer (Anstalt für Sudetendeutsche Heimatforschung) Reichenberg, 11 (1), 23–27. |
| Świerczewska-Gładysz, E., & Jurkowska, A. (2013). Occurrence and paleoecological significance of lyssacinosid sponges in the Upper Cretaceous deposits of southern Poland. Facies, 59, 763-777. |
| Tröger, K. A., & Christensen, W. K. (1991). Upper Cretaceous (Cenomanian-Santonian) inoceramid bivalve faunas from the island of Bornholm, Denmark. Danmarks Geologiske Undersøgelse, 28, 7-45. |
| Waldman M. (1965) A coelacanth, Macropoma, from the Chalk of Wiltshire, Proceedings of the Bristol Naturalists' Society, 111-112. |
| Wagner, W. (1963). Die Schwammfauna der Oberkreide von Neuburg (Donau). Palaeontographica Abteilung A, 166-250. |
| Wilmsen, M., Niebuhr, B., & Wood, C. J. (2001). Early Cenomanian (Cretaceous) inoceramid bivalves from the Kronsberg Syncline (Hannover area, Lower Saxony, Germany): stratigraphic and taxonomic implications. Acta Geologica Polonica, 51(2), 121-136. |
| Woods, H. (1911). A Monograph of the Cretaceous Lamellibranchia of England. Vol. II. Part VII. Inoceramus. Pages 261–284; Plates XLV–L. Monographs of the Palaeontographical Society, 64(314), 261-284. |
| Woods, M. A. (2002). The macrofossil biostratigraphy of the Turonian and Coniacian (Upper Cretaceous, Chalk Group) of southeast Devon. Proceedings of the Geologists' Association, 113(4), 333-344. |
